# Metabolic flux analyses to assess the differentiation of adult cardiac progenitors after fatty acid supplementation

**DOI:** 10.1016/j.scr.2019.101458

**Published:** 2019-07

**Authors:** Sophia Malandraki-Miller, Colleen A. Lopez, Rita Alonaizan, Ujang Purnama, Filippo Perbellini, Kathy Pakzad, Carolyn A. Carr

**Affiliations:** a Department of Physiology, Anatomy, and Genetics,Sherrington Building, University of Oxford, Oxford, UK; b National Heart and Lung Institute, Imperial College London, London, W12 0NN, UK

**Keywords:** Cardiac stem cells, Differentiation, Metabolism, Oleic acid OA, Fatty acid oxidation, PPARα, A.A, Ascorbic Acid, BSA, Bovine Serum Albumin, CKIT, proto-oncogene Ckit / tyrosine-protein kinase Kit (CD117), CDM, Cardiac Differentiation Medium, CPC, Cardiac Progenitor Cell, CX43, Connexin 43, DIFF, Differentiation samples, DIFF + OA, Differentiation samples + Oleic Acid, DMSO, DiMethylSulfOxide, EB, Embryoid Body, FA, Fatty Acid, FBS, Fetal Bovine Serum, FLK1, Fetal Liver Kinase 1 / (KDR) (Type III Receptor Tyrosine Kinase), HF, Heart Failure, HRPT, Hypoxanthine-guanine phosphoribosyltransferase, GATA4, GATA binding protein 4, GLUT1, Glucose transporter type 1, GLUT4, Glucose transporter type 4, ISL-1, ISL1 transcription factor/ (Insulin gene enhancer protein – Islet1), mESC, mouse Embryonic Stem Cells, MEF2C, Myocyte Enhancer Factor 2C, MI, Myocardial Infarction, MYH7, myosin, heavy chain 7, cardiac muscle, beta, NKX2.5, NK2 homeobox 5, OA, Oleic Acid (unsaturated fatty acid), OCT-3/4, Octamer-binding Transcription factor 4 / (POU5F1), Px, Passage x (x = number), PDK4, Pyruvate Dehydrogenase Kinase-4, PGC-1Α, Peroxisome Proliferator-Activated Receptor Gamma Coactivator 1, PFA, Paraformaldehyde, PPARΑ, Peroxisome Proliferator Activated Receptor Alpha, RXR, Retinoid X Receptor, SCA1, Stem cells antigen-1, SDHA, Succinate Dehydrogenase complex, subunit A, TERT, telomerase reverse transcriptase, TGF-β1, Transforming Growth Factor Beta 1, TNNT2, (Cardiac) Troponin T2

## Abstract

Myocardial infarction is the most prevalent of cardiovascular diseases and pharmacological interventions do not lead to restoration of the lost cardiomyocytes. Despite extensive stem cell therapy studies, clinical trials using cardiac progenitor cells have shown moderate results. Furthermore, differentiation of endogenous progenitors to mature cardiomyocytes is rarely reported.

A metabolic switch from glucose to fatty acid oxidation occurs during cardiac development and cardiomyocyte maturation, however *in vitro* differentiation protocols do not consider the lack of fatty acids in cell culture media. The aim of this study was to assess the effect of this metabolic switch on control and differentiated adult cardiac progenitors, by fatty acid supplementation.

Addition of oleic acid stimulated the peroxisome proliferator-activated receptor alpha pathway and led to maturation of the cardiac progenitors, both before and after transforming growth factor-beta 1 differentiation. Addition of oleic acid following differentiation increased expression of myosin heavy chain 7 and connexin 43. Also, total glycolytic metabolism increased, as did mitochondrial membrane potential and glucose and fatty acid transporter expression.

This work provides new insights into the importance of fatty acids, and of peroxisome proliferator-activated receptor alpha, in cardiac progenitor differentiation. Harnessing the oxidative metabolic switch induced maturation of differentiated endogenous stem cells.

(200 words)

## Highlights

• Culture of CPCs with oleic acid (OA) upregulates glycolysis and glucose oxidation.
• TGF-β differentiation of CPCs increases glycolytic flux.
• OA increases glycolysis, glucose and fatty acid oxidations of differentiated CPCs.
• The PPARα /PGC1α axis gets upregulated after oleic acid supplementation.

## Graphical abstract

**Figure:**
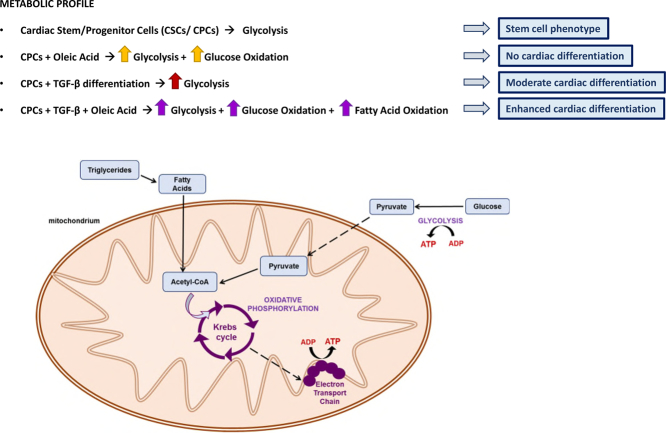

## 1 Introduction

**Fig. S1:**
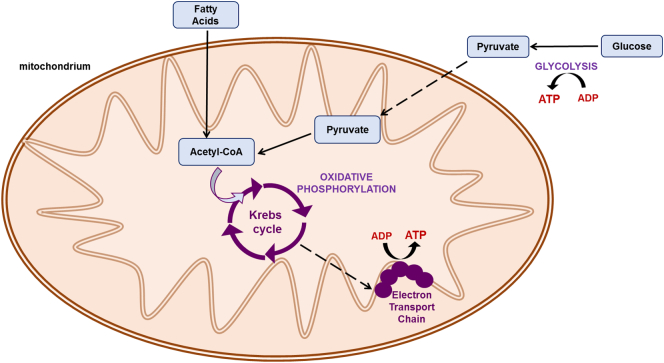
Schematic of basic cell metabolic pathways for energy production.

The heart uses a variety of substrates for energy production, while an array of metabolic networks guides adenosine triphosphate (ATP) production rates (Evans and Heather, 2016)^,^(Taegtmeyer et al., 2016). ATP can be produced *via* glycolysis (non-oxidative breakdown of glucose) (DeBerardinis et al., 2007), alternatively, pyruvate can enter the mitochondria and be metabolised *via* oxidative phosphorylation (Madeira, 2012) (Fig. S1).

A well-studied metabolic switch characterises the transition from cardiac stem cells to cardiomyocytes (CMs), highlighted by a shift from anaerobic to oxidative metabolism (Kolwicz et al., 2013), which allows for the increased yield of ATP to sustain contraction (Schönfeld et al., 1996). Another metabolic shift is also experienced by cardiac progenitor cells (CPCs) during transplantation *in vivo*, since CPCs are transferred from the culture medium, which can contain between 5.5 mM – 25 mM glucose and no fatty acids (FAs), to substrates in plasma that vary substantially. In mice, glucose levels have been measured between ~3.4–9.6 mM (Wolfensohn and Lloyd, 2003), while FAs in the range of ~0.18–0.6 mM (Perbellini et al., 2014).

In addition, both oleic acid (OA) and Transforming Growth Factor-Beta 1 (TGF-β1) are naturally found in the body, so in the context of cell therapy it is necessary to understand their effect on the transplanted cells. FAs (including oleic, palmitic, linoleic, and arachidonic acid) are natural ligands for Peroxisome Proliferator-Activated Receptor α (PPARα) (Göttlicher et al., 1992). The PPARα signalling pathway comprise a group of nuclear receptor proteins (Nuclear Receptors Nomenclature, C, 1999), and their target genes are involved in fatty acid (Gulick et al., 1994) and glucose metabolism (Finck et al., 2002). Ding et al. showed that PPARα levels were upregulated during differentiation of Embryonic Stem Cells (ESCs) to beating CMs, and that PPARα inhibition prevented differentiation (Ding et al., 2007a). Furthermore, PPAR co-activator 1α (PGC-1α) (Sanderson et al., 2009), is involved in metabolic processes (Handschin and Spiegelman, 2006) including regulation of genes involved in FA oxidation (Vega et al., 2000). Interestingly, a study conducted in 2014 on the HepG2 hepatic cell line, showed that not all FAs have the same binding affinity to PPAR-alpha and therefore, do not necessarily result in its activation. While OA and palmitoleic acid increased PPRAα transactivation, palmitate and stearic acid inhibited it (Popeijus et al., 2014).

Various strategies have been applied to develop the optimal protocol for *in vitro* cardiac differentiation of stem cells (Malandraki-Miller et al., 2018). Goumans et al. used TGF-β1 for differentiation of human adult atrial stem cell antigen-1-positive (Sca1^+^) CPCs (Goumans et al., 2008; Smits et al., 2009).

Recently, there have been studies exploring metabolic manipulation and the subsequent effect on cell differentiation and maturation, in hPSC-CMs. In 2017, Correia et al. investigated how different substrates affect the functional maturation of hPSC-CMs (Correia et al., 2017). By testing different media compositions, they demonstrate that culture medium supplemented with fatty acids and galactose, lacking glucose, forced the cells to shift to oxidative phosphorylation. That resulted in a phenotype more similar to human adult CMs than previously reported. In addition, Hu et al. investigated the mechanism that links metabolism and the maturation of hPSC-CMs, showing that reliance on glucose and the HIF1α-LDHA axis are involved in the immature phenotype of hPSC-CMs (Hu et al., 2018). They afterwards proved that metabolic shift towards oxidative metabolism led to maturation of the hPSC-CMs. Hitherto, to the best of our knowledge, no cardiac differentiation protocol has investigated the effect of FA supplementation on adult CPCs' metabolism and differentiation.

On the basis of the link between metabolic state and cell phenotype, we suggest that manipulating substrate availability *in vitro* could trigger differentiation of CPCs. In addition, the different layers of regulation that PPARα and PGC-1α exert on differentiation and metabolism lead us to hypothesise that stimulating this axis could enhance cell differentiation. Here we reveal the effect of fatty acid (OA) availability, in culture, on the metabolic maturation of adult CPCs. We also elucidate the effect that OA has on control and differentiated CPCs.

## 2 Materials & methods

### 2.1 Animals

Male C57BL/6 mice (Harlan, Oxon, UK) were housed in a 12-h light–dark cycle, controlled temperature and humidity, with water and food *ad libitum*. All animal procedures were reviewed and approved by the University of Oxford Animal Welfare and Ethical Review Board and conforms to the Animals (Scientific Procedures) Act 1986 incorporating Directive 2010/63/EU of the European Parliament.

### 2.2 Isolation and expansion of mouse cardiac progenitor cells (CPCS)

Mouse atria were excised, washed with Dulbecco's phosphate buffered saline (DPBS) (Invitrogen, Fisher Scientific – UK Ltd) containing Penicillin / Streptomycin (P/S) and minced mechanically. The tissue-pieces were transferred in 0.5 ml of the digestion solution (comprising of 0.1% trypsin and 0.1% Collagenase II (Calbiochem, 286 U/ mg), incubated in a shaking water bath at 37 °C for 1 h. Every 10′ the digestion-mix was mechanically triturated, the supernatant was collected, neutralized with CPC medium and plated on fibronectin-coated 6-well plates. CPC medium comprised of Iscove's modified Dulbecco's medium (IMDM) (Invitrogen, Fisher Scientific – UK Ltd) with 20% foetal bovine serum (FBS) (Invitrogen, Fisher Scientific – UK Ltd), 1 U/ ml penicillin, 1 μg/ ml streptomycin and 0.2 mM l-glutamine (P/S/G), (Gibco, Life Technologies, Fisher Scientific – UK Ltd). Fresh digestion solution was added to the remaining tissue pieces as before, until 1 h was reached. This digestion produced surviving fibroblast-like cells, named “CPCs”, attaching after 2 days. The CPCs were passaged when they reached 90% confluency, for further experiments and analysis.

### 2.3 RNA extraction, reverse transcription &qPCR reaction

Total RNA was extracted from frozen cell pellets, using the RNeasy Mini Kit (QIAGEN Ltd. – Manchester UK), following the manufacturer's protocol. Complementary DNA was synthesized, using the High Capacity cDNA Reverse Transcription kit (Life Technologies, Fisher Scientific – UK Ltd), following the manufacturer's protocol. cDNA aliquots were kept at −20 °C for further use. qPCR was performed using the StepOnePlus Real-Time PCR System (Applied Biosystems, Life Technologies, Fisher Scientific – UK Ltd). Relative mRNA levels were normalised to Sdha and Hrpt housekeeping genes. As detection system for the reaction SYBR green fluorescent intercalating dye was used (SYBR Green PCR mastermix, Life Technologies, Fisher Scientific – UK Ltd). For primer information see Supplementary material. Data was analysed using the published ΔΔCt method Livak method (Livak and Schmittgen, 2001), plotting the data as 2^−ΔΔCt±SE^.

### 2.4 Immunocytochemistry

CPCs P4 were seeded in 24-well plates, on fibronectin-coated cover slips (2 × 104 cells / well) and were fixed with 4% paraformaldehyde for 30′ at 4 °C. For intracellular proteins, cells were permeabilised with 0.2% Triton X for 10′. Then cells were blocked with a 2% FBS and 2% bovine serum albumin (BSA, Sigma-Aldrich Ltd. – Dorset, UK) solution for 30′. Primary antibody staining followed overnight at 4 °C. After washing with PBS, when required, cells were labelled with secondary antibody (30′, room temperature). Afterwards samples were co-stained with 0,1% 4′,6-diamidino-2-phenylindole (DAPI, Sigma-Aldrich Ltd. – Dorset, UK), and mounted on glass slides using a 50% PBS and 50% Glycerol (Fisher Scientific – UK Ltd) solution. Slides were kept at 4 °C protected from light, until imaging. Immunostaining was assessed using light confocal microscopy (Inverted Olympus Fluoview FV1000 Confocal system). For image analysis FIJI software (“Fiji Is Just ImageJ”) was used. Positive staining was assessed compared to a negative control; either cells stained only with 4′,6-diamidino-2-phenylindole (DAPI) or with just the secondary antibody and DAPI. For antibody information see Supplemental material.

### 2.5 Mitochondrial staining

For mitochondrial imaging, the MitoTracker® Red CMXRos (Fisher Scientific – UK Ltd) intracellular dye was used, following manufacturer's protocol. CPCs at P4 were incubated for 40’with 10 nM MitoTracker in non-FBS medium. After washing with DPBS, they were fixed with 4% paraformaldehyde and imaged using light confocal microscopy. The average fluorescence intensity was analysed using the FIIJI image analysis software.

### 2.6 Mouse-esc differentiation – Embryoid bodies

The differentiated mouse ESCs, which were used as a positive control for comparison with differentiated CPCs in RT-PCR experiments, were donated by Dr. Richard Tyser (Prof. Paul Riley group, DPAG, Oxford). The samples were collected either at day 4, day 7,or day 14 of differentiation. Differentiation was induced using the hanging drop culture – embryoid body formation method (Boheler et al., 2002) (EBs d4, EBs d7, EBs d14).

### 2.7 TGF-β1 differentiation

Cardiac differentiation was induced using TGF-β1 (Smits et al., 2009). CPCs were seeded in CPC medium on gelatine-coated flasks 0,1% (Sigma-Aldrich Ltd. – Dorset, UK); at a density of 25 × 10^4^ cells per 25 cm^2^ flask or at 2 × 10^4^ cells per well in a 24-well plate. The next day, the medium was replaced with Cardiac Differentiation Medium (CDM) (47% IMDM, 47% Ham's F12 – GlutaMAX-I, 2% Horse serum, 1% MEM non-essential amino acids, 1% Insulin-Transferrin-Selenium, 2% Pen/Strep, containing 5 μM 5-Azacytidine. 5-Azacytidine was also added at the next 2 days, and then the medium was refreshed. From day 6 forward, 1 mM Ascorbic Acid (AA) was added every 2 days and 1 ng/ ml TGF-β1 twice weekly, while medium was refreshed every 2 days. The cells were collected after 25 days of differentiation for analysis. Undifferentiated, but over-confluent, cells were used as a control.

### 2.8 Oleic acid supplementation

CPCs P4 were seeded in 6-well plated for either 48 h, 1 week or 1 month, with the CDM basal medium (without any drug addition) supplemented with 75 μM, 150 μM and 300 μM OA conjugated to BSA. As a control, BSA supplemented CDM was used. The media was replenished every 2 days in all conditions.

Following the 25-day TGF-β1 differentiation, as described above, the differentiating cells were cultured in OA 300 μM-supplemented CDM, for either 1 week or 1 month.

### 2.9 ^14^C-glucose oxidation measurement

Glucose oxidation was measured using the method of the Collins et al. (Wolfensohn and Lloyd, 2003) with some modifications. CPCs were seeded on gelatine-coated 24-well plates (20 × 10^4^ cells/ well) and were grown in basal CDM medium as untreated “control” condition (CNTRL), differentiated or treated with OA for 48 h or 1 week. Subsequently, they were incubated for 4 h in no-glucose DMEM (A14430, Gibco, Life Technologies, Fisher Scientific – UK Ltd) supplemented with 12 mM glucose containing 0.185 MBq D-U-^14^C-glucose (0.5 ml/ well). DMEM-alone was used as a negative CNTRL and wells without cells containing DMEM with ^14^C-glucose were used to measure background radioactivity. A 24-well plate formed the lid of the apparatus with a perforated rubber gasket, with holes corresponding to each well, separating the two plates. The ^14^CO_2_ produced by the glucose oxidation was trapped on KOH-soaked filter papers in the upper wells. To trigger the release of the ^14^CO_2_, perchloric acid was added to the well, at each desired time-point, to kill the cells. Subsequently, the samples were kept for 1 h to allow for the release of dissolved ^14^CO_2_. Filter papers containing trapped ^14^CO_2_ were analysed using a scintillation counter, and the radiation counts per minute (CPM) were measured.

### 2.10 ^3^H palmitate oxidation

CPCs were seeded in gelatine-coated 24-well plates (20 × 10^4^ cells/ well) and were grown in basal CDM medium, as untreated CNTRL, differentiated or treated with OA for 48 h and 1 week. Subsequently the cells were incubated in no-glucose DMEM medium that was supplemented with 12 mM glucose, 300 μM palmitate, conjugated to 20 μM albumin and 0.022 MBq ^3^H-palmitate for 6 h. Palmitate oxidation rates were determined by the production of ^3^H_2_O from the mitochondria (Barr and Lopaschuk, 1997; Belke et al., 1999). Perfusate aliquots contained both ^3^H_2_O and ^3^H-palmitate, so the ^3^H_2_O was separated *via* Folch extraction. 1.88 ml of chloroform:methanol (1:2 *v*/v) solution, 625 μl chloroform and 625 μl KCl-HCl solution (2 M KCl, 0.4 M HCl) were added to 0.5 ml of the perfusate sample. The solution was rotated on a laboratory Stuart rotator SB3 at 40 rpm for 1 h before removing the top aqueous layer, while the organic layer was discarded. A solution of 1 ml choloform, 1 ml methanol and 0.9 ml KCl-HCl was added to the extracted aqueous solution, before further rotation for 1 h at 40 rpm. The top aqueous layer was once again removed and retained, and radioactivity was counted in counts per minute (CPM) using a scintillation counter. 0.5 ml medium at time 0 was used as a control to determine the specific activity of the buffer. For each aliquot sample, palmitate oxidation rates were calculated (Barr and Lopaschuk, 1997).

### 2.11 ^3^H glucose measurement of glycolytic flux

CPCs were seeded in gelatine-coated 24-well plates (20 × 10^4^ cells/ well) and were grown in in basal CDM medium, as an untreated CNTRL, differentiated or treated with OA for 48 h and 1 week. Subsequently, they were incubated for 6 h in no-glucose basal DMEM supplemented with 12 mM glucose containing 0.118 MBq ^3^H-glucose. Glycolytic rates were determined through the conversion of ^3^H -glucose to ^3^H_2_O *via* enolase which converts 2-phosphoglycerate to phosphoenolpyruvate and releases H_2_O as a by-product. The samples contained both ^3^H_2_O and ^3^H-glucose. Therefore, the ^3^H_2_O was separated from the ^3^H-glucose using a Dowex 1 × 4 chloride form 100–200 mesh (Sigma, UK) anion exchange column. 250 g of Dowex resin was added to a 1.25 M NaOH and 1.61 M boric acid solution, and washed with distilled H_2_O until pH < 7.5. Dowex resin was added to glass Pasteur pipettes plugged with glass wool and the columns were washed with distilled H_2_O and allowed to drain. Then 200 μl of sample was added to each column allowing for the ^3^H -glucose to bind to the column for 15′, and ^3^H_2_O to be eluted into scintillation vials. Distilled H_2_O (2 ml) was added into the column to wash down any residual samples. Sample radioactivity was counted in counts per minute (CPM) using a scintillation counter.

### 2.12 Cell viability

To assess cell viability after Oleic Acid supplementation in the cell medium, treated CPCs were washed with PBS, trypsinised, and then counted using trypan blue stain, using both the standard haemocytometer approach and a Countess Automated Cell Counter (Invitrogen, Fisher Scientific – UK Ltd).

### 2.13 Statistical analysis

For all experiments (except for the case of mESCs) the ‘n’ number refers to biological replicates, that represent cells originating from different mice, and each tested once. The ‘n’ number for the EB mESCs represents technical replicates, of the donated cell line samples. Results are presented as means ±SE for qPCR and metabolic flux analyses, and means ±SD for other analyses. Differences were considered significant at *p* < .05, determined using analysis of variance with a Student's *t*-test. Experiments with >2 test groups were assessed by a one-way analysis of variance (ANOVA) with a Tukey post-hoc test (GraphPad Prism 7).

## 3 Results

### 3.1 TGF-β1 differentiation increases expression of TNNT2 in mouse adult CPCs

**Fig. 1:**
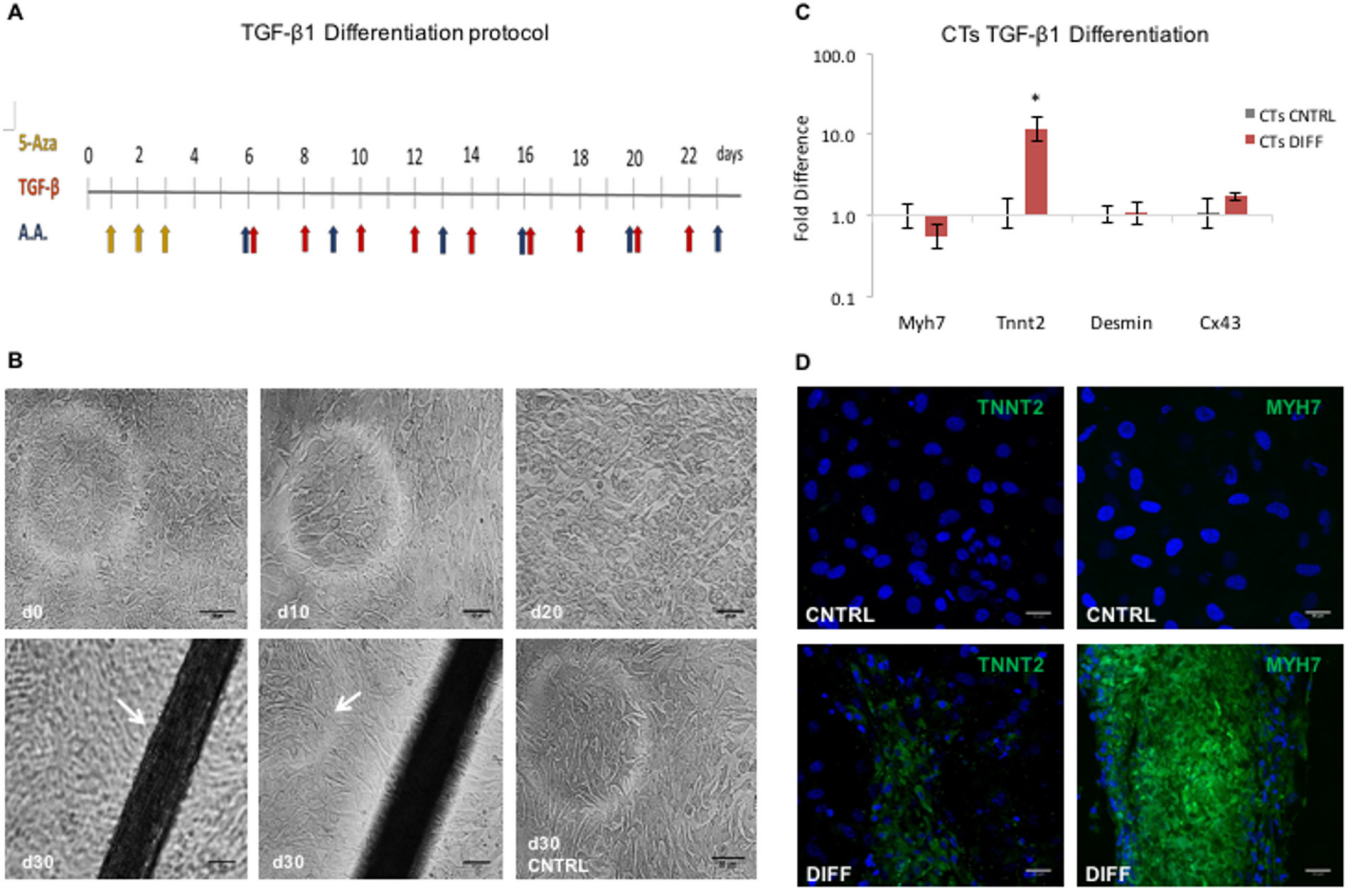
Differentiation of CPCs: (A) Schematic representation of the timeline of the TGF-β1 differentiation protocol; yellow arrows mark the days when 5-Aza was added, blue is for Ascorbic Acid and red for TGF-β1. (B) Cell morphology of differentiating CPCs, at day 0, 10, 20 and 30, under light microscope. Arrow at d30 left image: indicating the tubular structure, at d30 right image: indicating the cell layer underneath the tubular structure (scale bars: 100 μm). (C) qPCR of differentiating CPCs normalised to the respective undifferentiated control samples (*n* = 4, *p < .002, error bars: standard error). (D) Differentiated CPCs, expression of MYH7 and TNNT2 shown in green; a,b: control samples, c,d: differentiated samples. (blue: DAPI, scale bars: 30 μm). (For interpretation of the references to colour in this figure legend, the reader is referred to the web version of this article.)

**Fig. S2:**
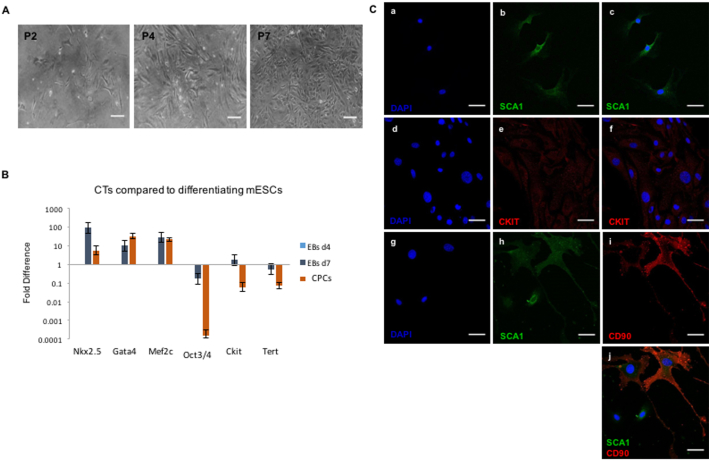
Characterisation of CPC cells. A) CPCs under phase contrast microscope, as observed at (a) p2, (b) p4, and (c) p7. (scale bars 100 μm). B) Gene expression levels of stemness and cardiac lineage markers in CPCs at P4, compared to mESCs differentiating to CMs at d4 and d7 of differentiation (*n* = 3). C) Immunocytochemistry on CPCs, (a-c) SCA1 (d-f), CKIT, (g-i) CD90/SCA1 double staining, with DAPI (a, d, g), (k) secondary antibodies for negative control. Scale bars: 50 μm.

To assess the differentiation potential of the CPCs (for CPC characterisation profile see Supplementary Material and Fig. S2), the TGF-β1/5-Azacytidine/Ascorbic Acid (A.A.) protocol was used (Fig. 1A). The differentiating CPCs (“DIFF”) were monitored throughout, and at day 30 formed tubular structures (Fig. 1B). Gene expression analysis *via* qPCR showed a significant increase in cardiac troponin-T 2 (TnnT2) levels (Fig. 1C). The presence of TNNT2 and myosin heavy chain β (MYH7) was confirmed by immunocytochemistry, with the latter being confined to the organoids (Fig. 1D).

### 3.2 Stages of mESC differentiation *VIA* embryoid body formation

**Fig. 2:**
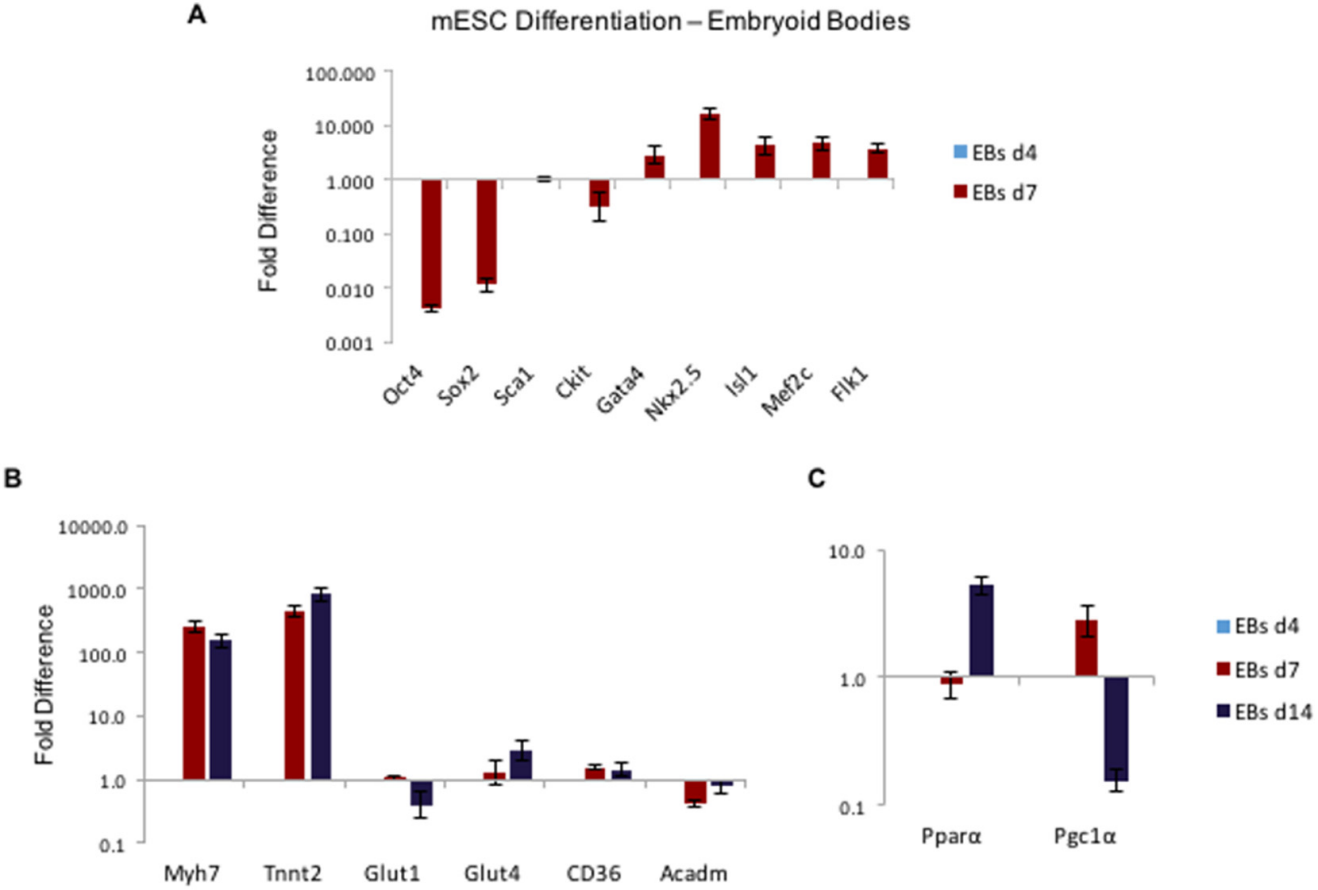
Gene expression in differentiating ESCs: (A) Gene expression levels of stemness markers (Oct4, Sox2, Sca1, Ckit), early-onset cardiac (Gata4, Mef2c, Nkx2.5, Isl1) and endothelial (Flk1) markers in mESCs at d4 and d7 of differentiation of ESCs to CMs, normalised to EBs d4. (B) Gene expression levels of late-onset cardiac markers (TnnT2 and Myh7) and Glut1, Glut4 in mESCs at d4, d7 and d14 of differentiation to CMs, normalised to EBs d4. (C) Gene expression levels of PPARα and Pgc1α in mESCs at d4, d7 and d14 of differentiation to CMs, normalised to EBs d4 (*n* = 3).

To characterise the metabolic changes we should expect to see during *in vitro* differentiation, a differentiating mESC line was used as a positive control. Agreeing with the anticipated genetic changes, the stemness genes (Oct4, Sox2, Sca1, Ckit) showed reduced expression as mESCs progressed from day 4 (EBs d4) to day 7 (EBs d7) of differentiation, while the early-onset cardiac markers (Gata4, Mef2c, Nkx2.5, Isl1) increased, as did the endothelial marker Flk1 (Fig. 2A). The gene expression of late-onset cardiac markers (Tnnt2 and Myh7) increased from day 4, through day 7 to day 14 (EBs d14) of differentiation. Expression of the insulin-sensitive glucose transporter Glut4 and the fatty acid transporter CD36 increased, while that of the glucose transporter Glut1, predominant at the fetal stage, was reduced (Fig. 2B). PPARα expression was unchanged from day 4 to day 7 of embryoid body differentiation and then showed an 8-fold increase. Pgc-1α was upregulated on day 7 and then downregulated when the cells were terminally differentiated (Fig. 2C).

### 3.3 Oleic acid supplementation stimulates the PPARα pathway in mouse adult CPCs

**Fig. 3:**
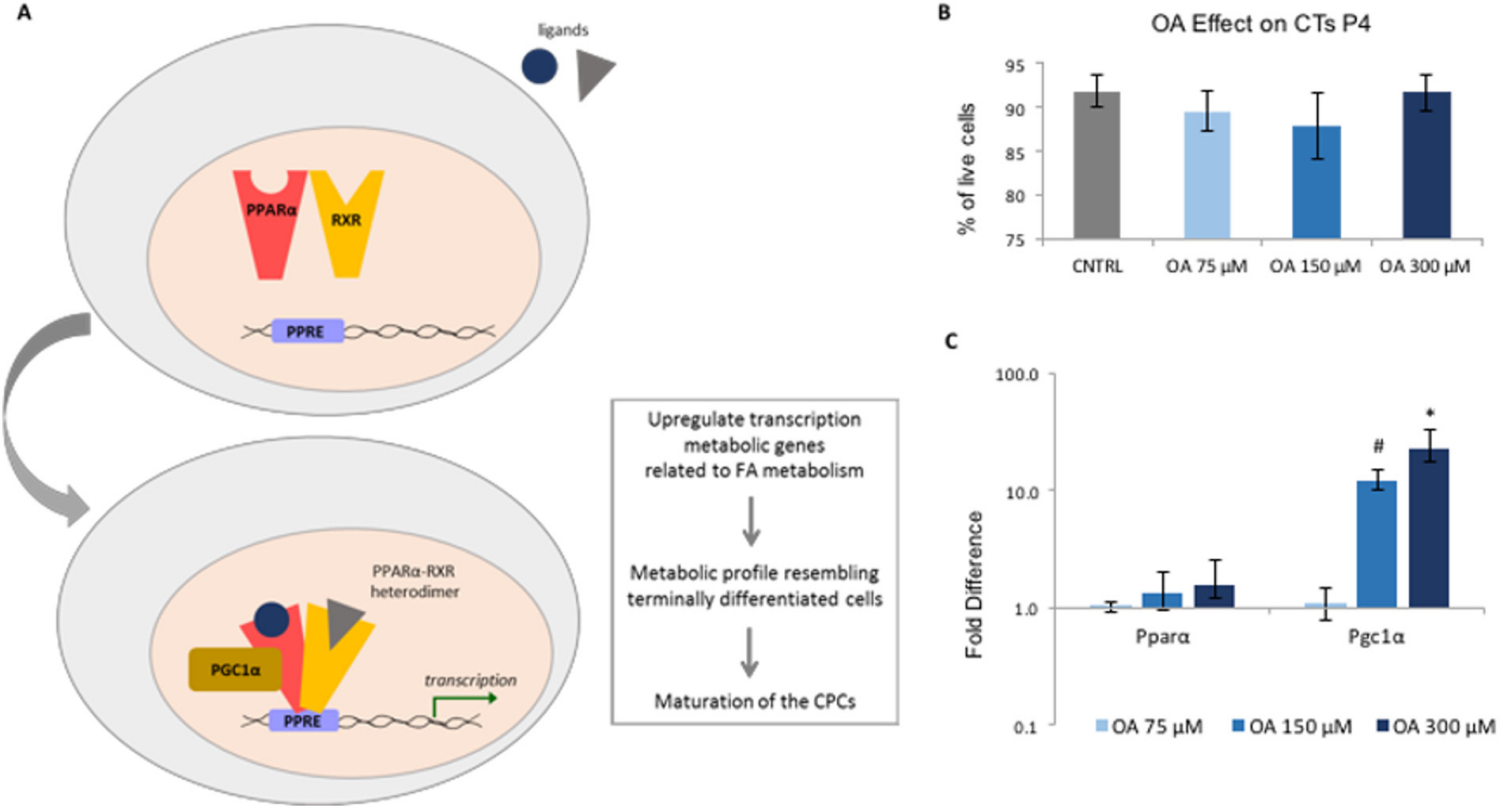
OA stimulation of the PPARα pathway: (A) Schematic representation of the PPARα/PGC-1α axis: PPARα binds to RXR, the heterodimer is activated by ligands and PGC-1α acts as a co-activator for PPARα. The heterotrimer binds to the PPARα response element (PPRE), in the promoter of target genes. (B) The effect of 75 μM, 150 μM or 300 μM of OA on cell viability after 1 week of incubation. (C) Effect of 48 h supplementation with OA at increased concentrations. Effect of OA 75 μM, 150 μM, 300 μM on the expression of PPARα, PGC-1α in P4 CPCs, normalised to OA 75 μM. (n = 4, *p < .02, #*p* < .01, indicating difference to OA 75 μΜ; error bars: standard error).

**Fig. S3:**
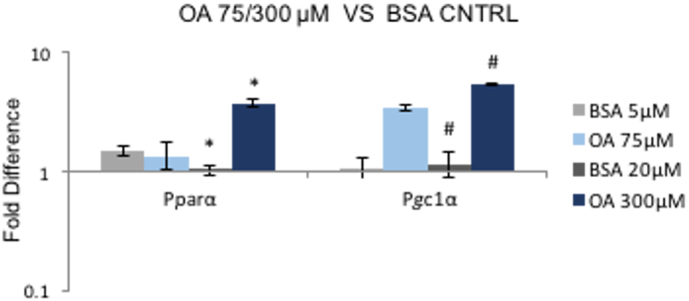
OA effect on PPARα / PGC-1α compared to the respective BSA control. Effect of 48 h supplementation with OA 75 μM and 300 μM on the expression of PPARα, Pgc1α, on CPCs P4, normalised to 48 h BSA supplementation with 5 μM and 20 μM, respectively. (*n* = 4; error bars: standard error, *p < .04, #*p* < .03, indicating difference to the respective BSA CNTRLs). Table showing the corresponding BSA concentrations for eack OA concentration, respectively.

To investigate the effect of FAs as a cell culture substrate, the CPC culture medium was supplemented with OA. The effect of 75 μM, 150 μM or 300 μM of OA on cell viability was checked and was not found to be toxic (Fig. 3B). 300 μM was selected as the highest dose because high levels of FA have been shown to be cytotoxic (De Sousa Andrade et al., 2005; Haeiwa et al., 2014). PGC-1α expression was significantly upregulated as the concentration of OA increased, but PPARα expression did not change (Fig. 3C). Since the OA supplement was pre-conjugated with BSA, the gene expression of PPARα and PGC-1α was also compared to the untreated controls which had been supplemented with BSA, at the respective concentrations. Both PPARα and PGC-1α expression increased >5-fold with 300 μM of OA, compared to the BSA controls (Supplementary Fig. S3).

### 3.4 Oleic acid supplementation metabolically matures mouse adult CPCs

**Fig. 4:**
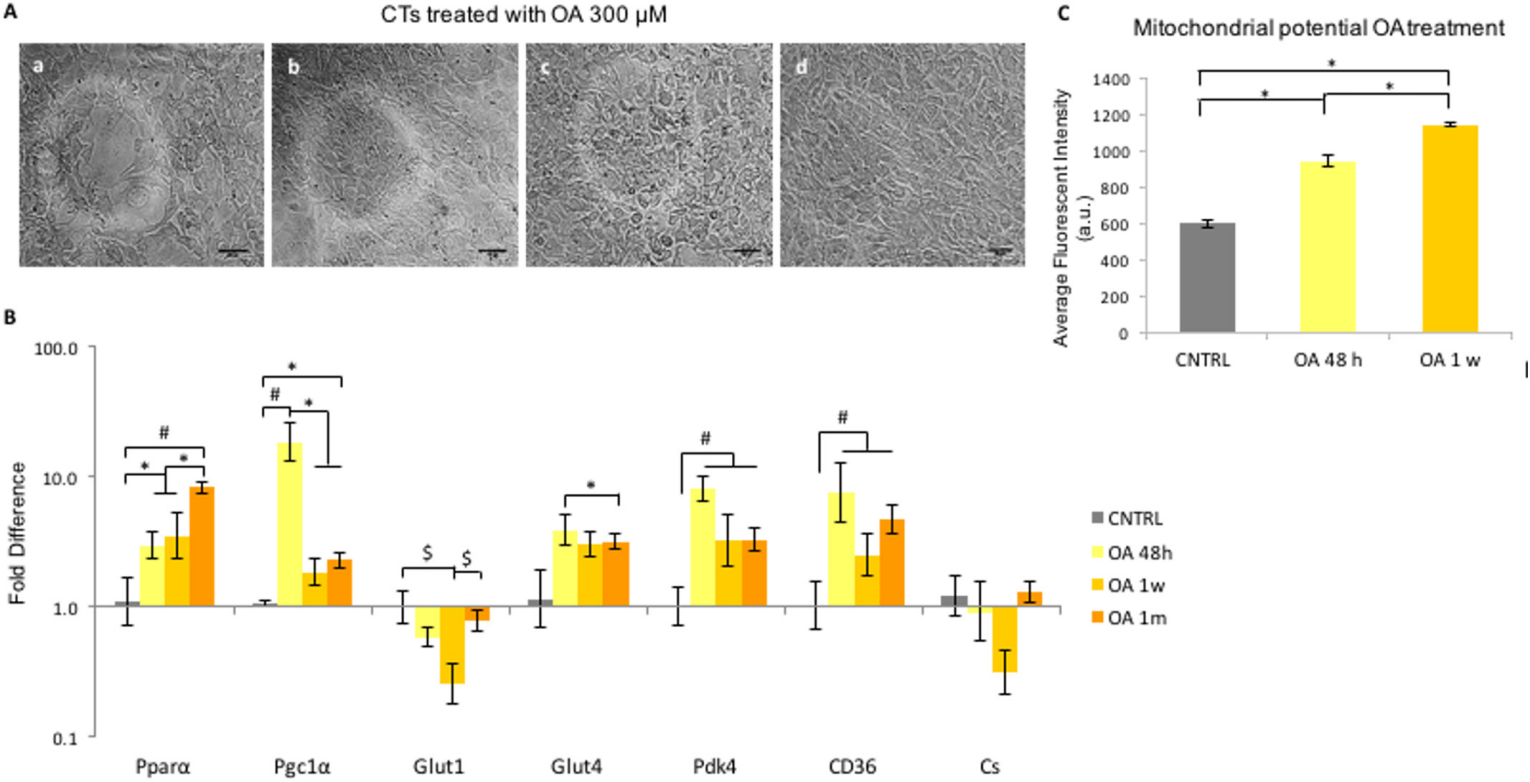
Long-term stimulation with OA: (A) P4 CPCs treated with OA for 48 h (4a), 1 week (4b) and 1 month (4c), as well as untreated CPCs grown for 1 month (scale bars: 100 μm). (B) Effect of 48 h, 1 week, 1 month supplementation of P4 CPCs with OA on the expression of metabolic genes, normalised to control untreated CPCs. (n = 3; error bars: standard error, *p < .05, #p < .02). (C) Effect of OA treatment for 48 h and 1 week, estimated as average fluorescent intensity, after staining with MitoTracker Red CMXRos red. *p < 0,00001 normalised to untreated CNTRL, and OA 48-h treatment, respectively. (For interpretation of the references to colour in this figure legend, the reader is referred to the web version of this article.)

To test the long-term effect of OA treatment on the metabolism of CPCs, gene expression levels were investigated after treatment of CPCs with OA 300 μM for 48 h, 1 week and 1 month. The CPC morphology was observed for whole period, during which cells kept proliferating and no cell death was observed (Fig. 4A).

PGC-1α gene expression was significantly upregulated after 48 h and then decreased (Fig. 4B). PPARα and Glut4 were also significantly upregulated with OA supplementation (Fig. 4B). Pdk4 significantly increased with OA addition at all time-points, as did the fatty acid translocase receptor CD36, which functions as an OA receptor (Fig. 4B). No change in gene expression of citrate synthase (Cs) was observed (Fig. 4B).

**Fig. S4:**
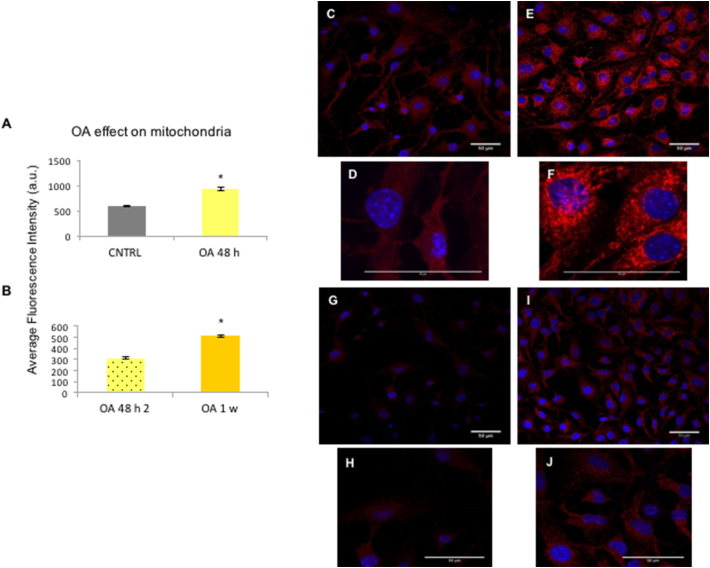
Mitochondrial staining after OA treatment: Short-term effect of OA in cell mitochondria. Two separate experiments were conducted; average fluorescence intensity of untreated control CPCs P4, compared to CPCs P4 treated with OA 300 μΜ for 48 h (A), and compared to CPCs treated for 1 week (B). Raw data; untreated CNTRL (C,D) and treated with OA 300 μΜ for 48 h (E,F left), stained with MitoTracker® Red CMXRos. Images D, E are taken after 5% zoom of each respective group. (scale bars: 50 μm). Long-term effect of OA in cell mitochondria; CPCs P4; treated with OA 300 μΜ for 48 h (G,H) or 1 week (I,J), stained with MitoTracker® Red CMXRos. Images H,J are taken after 2.5% zoom of each respective group. (scale bars: 50 μm)

To investigate mitochondrial activity, CPCs were incubated with MitoTracker® Red CMXRos. CNTRL untreated CPCs were compared to 48 h of OA treatment, and 1 week of OA supplementation (Fig. 4C). A significant increase in fluorescence was observed following prolongation of the OA treatment (Fig. 4C, Supplemental Fig. S4), suggesting that the mitochondria were more active, since the CMXRos dye accumulates in live cells based on the mitochondrial membrane potential (Cottet-Rousselle et al., 2011).

**Fig. 5:**
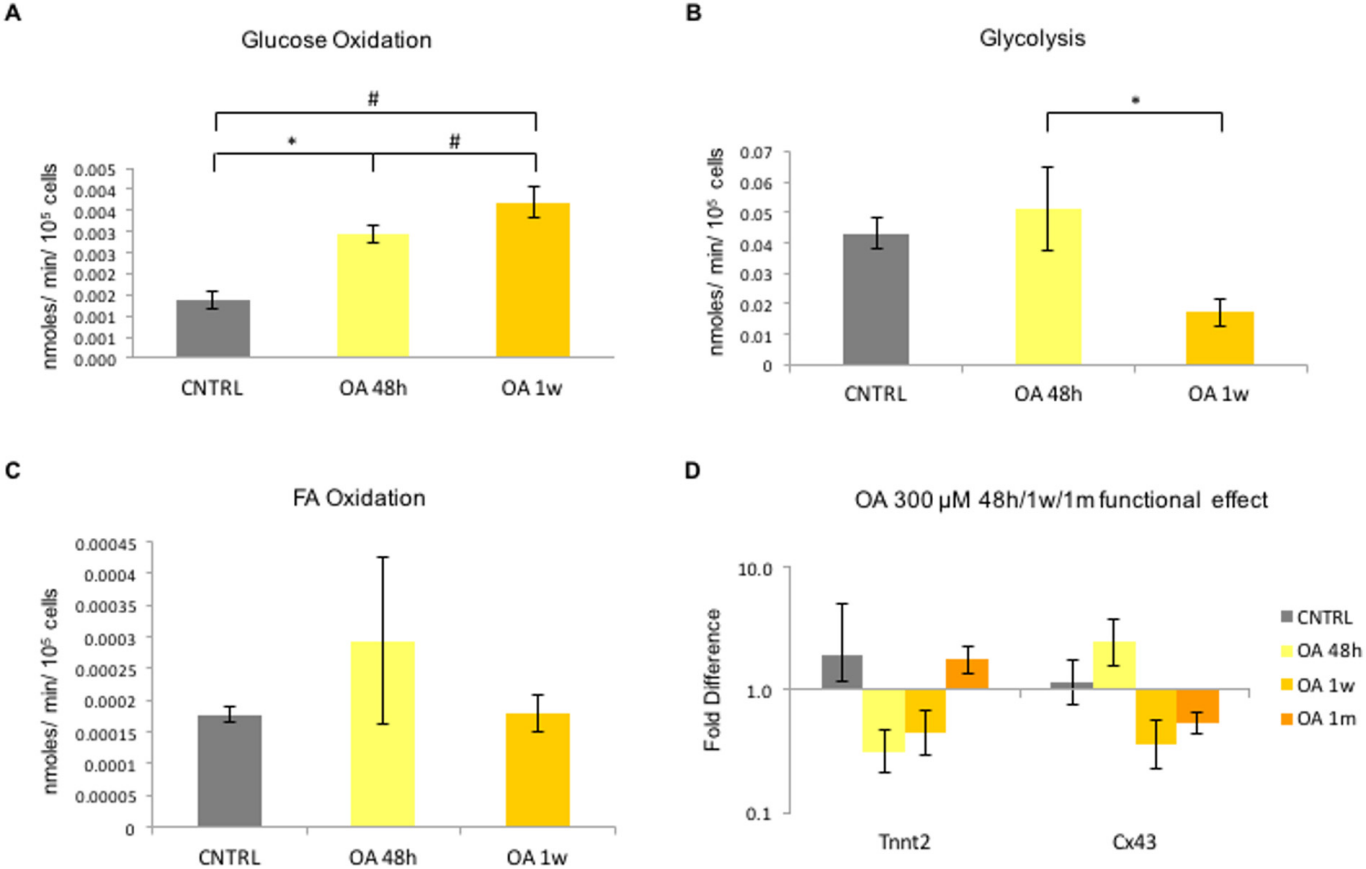
OA supplementation affects substrate metabolism: Effect of supplementation with OA on (A) glucose oxidation levels, (B) Glycolysis and (C) FA Oxidation levels, measured as nMoles/min/10^5^ cells, compared to control untreated CPCs. (n = 4; *p < .04, #p < .01 indicating differences amongst the groups, error bars: standard error); and D) on the expression of differentiation genes in P4 CPCs normalised to control untreated CPCs. (n = 3; error bars: standard error).

The level of substrate utilisation was assessed using radioactive tracer analysis. After supplementing the CPCs with OA for 48 h and 1 week, glucose oxidation was increased significantly (Fig. 5A), glycolytic flux was significantly reduced after 1 week of OA incubation (Fig. 5B), while FA oxidation remained unchanged (Fig. 5C). Despite these changes, qPCRanalysis showed no significant change in the gene expression of Tnnt2 or Cx43 after treatment with OA at any time point (Fig. 5D). Myh6 and Myh7 were not expressed under any of the conditions.

### 3.5 Oleic acid addition further matures differentiated CPCS functionally and metabolically

**Fig. 6:**
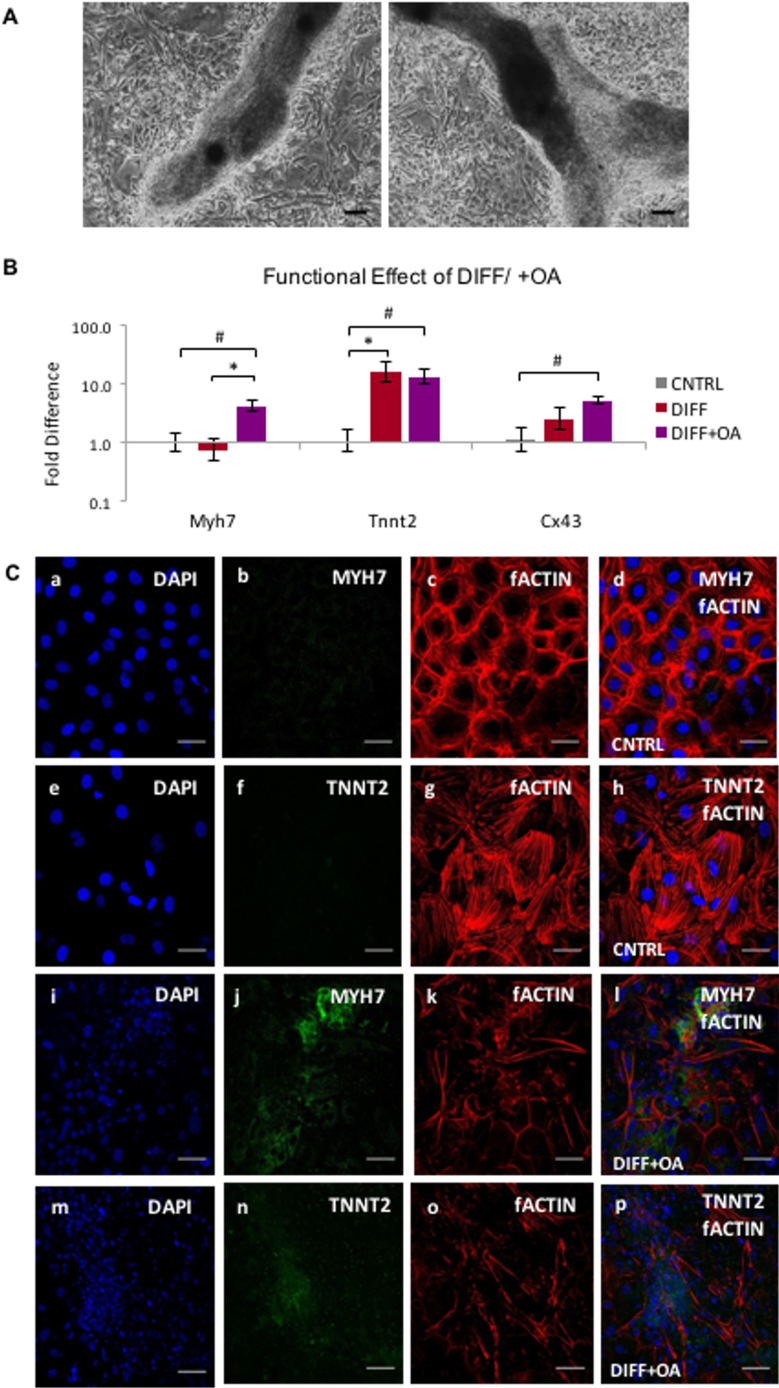
OA supplementation of differentiating CPCs: (A) Cell morphology of CPCs differentiated using the TGF-β1 protocol, followed by 1 week of OA 300 μM supplementation, under light microscope (scale bars: 100 μM). (B) Effect of OA treatment post-differentiation on CPCs, compared to non-treated differentiated CPCs and CNTRL undifferentiated P4 CPCs. (n = 4, *p < .02, $p < .01 compared to CNTRL, error bars: standard error). (C) Immunocytochemistry on control undifferentiated samples (top two rows) and differentiated samples with OA addition (DIFF+OA; bottom two rows). (MYH7 and TNNT2 shown in green, co-stained with DAPI in blue, scale bars: 50 μm). (For interpretation of the references to colour in this figure legend, the reader is referred to the web version of this article.)

Based on the data indicating upregulation of the PPARα pathway after OA treatment of CPCs, OA was used after TGF-β1 differentiation (“DIFF+OA”) to assess whether differentiated cells could be matured metabolically. The differentiating CPCs formed cell clusters as before (Fig. 6A), but with the OA treatment the cells surrounding them were smaller than in the non-treated samples. qPCR analysis of mature cardiac genes (Myh7, Tnnt2, Cx43) showed that both Myh7 and Cx43, which were unchanged after differentiation (Fig. 1C), were significantly upregulated after OA treatment. Tnnt2 gene expression did not increase further (Fig. 6B). Immunocytochemistry confirmed the expression of MYH7 and TNNT2 in the differentiated CPCs treated with OA (Fig. 6C). Filamentous actin (fActin) staining, to assess the shape and structural components of the cells, did not indicate any CM-like striations (Fig. 6C).

**Fig. 7:**
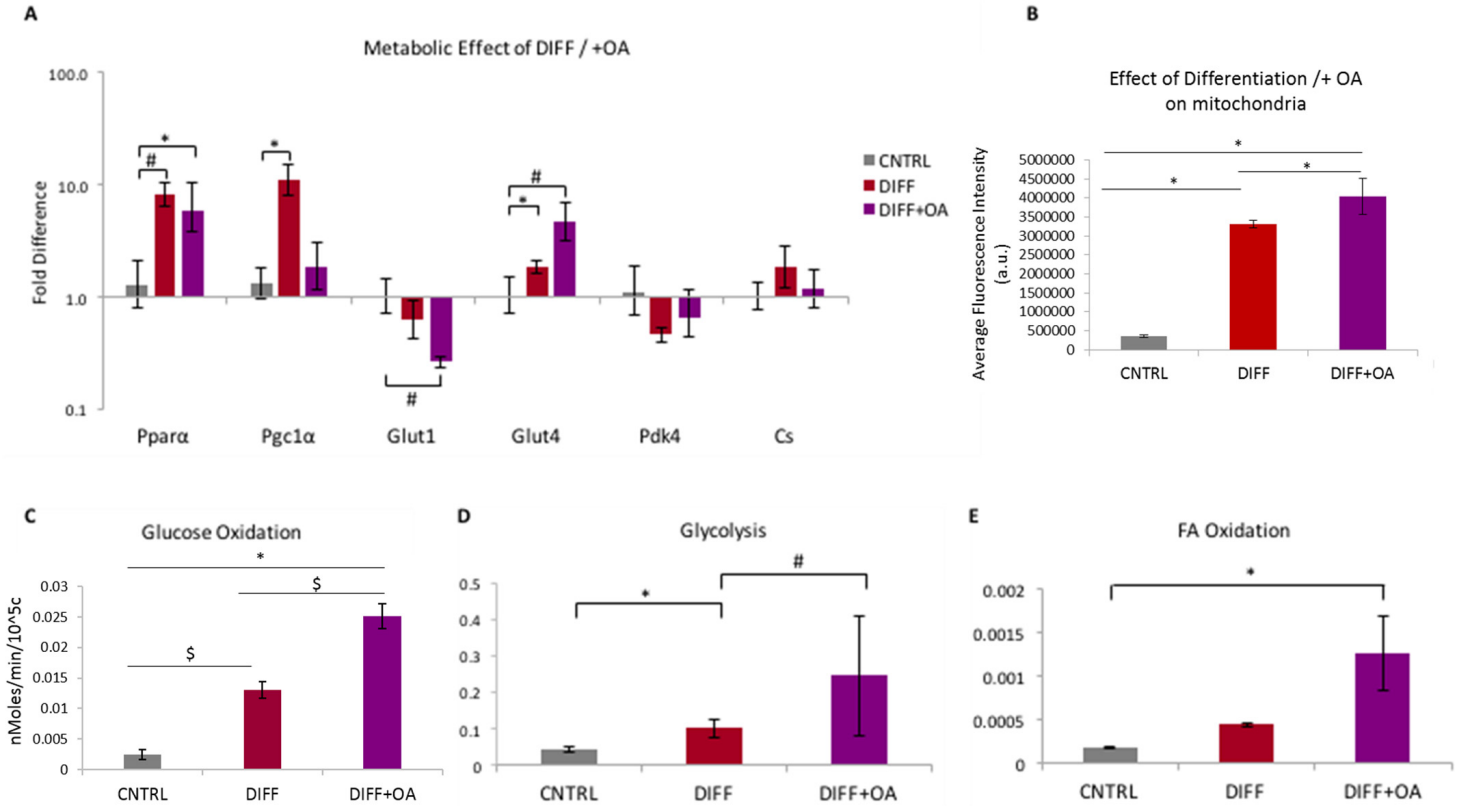
OA supplementation affects substrate metabolism in differentiated CPCs: (A) Metabolic effect of 1 week supplementation with OA post-differentiation on gene expression in CPCs, compared to non-treated differentiated CPCs and CNTRL undifferentiated CPCs. (n = 4, *p < 0,05, $p < 0,01 indicating difference to CNTRL, error bars: standard error). (B) Effect of differentiation, and differentiation followed by 1 week of OA 300 μM, on cell mitochondria, estimated as average fluorescent intensity, after staining with MitoTracker Red CMXRos red. (*n* = 12 cells, 3 replicates, *p < .001). (C) Effect of TGF-β1 differentiation, and differentiation followed by 1 week supplementation with OA, on the glucose oxidation levels, (D) Glycolysis, and E) FA Oxidation, measured as nMoles/ min/ 10^5^ cells, normalised to control untreated CPCs. (n = 3; *p < .03, #p < .01, $p < .001 indicating differences amongst the groups, error bars: standard error). (For interpretation of the references to colour in this figure legend, the reader is referred to the web version of this article.)

**Fig. S5:**
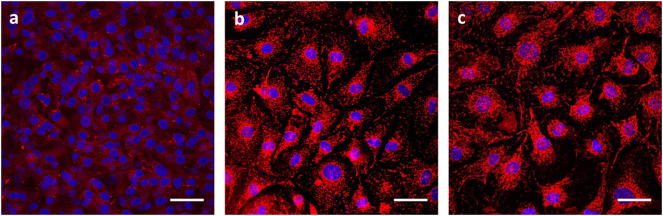
Mitochondrial staining after differentiation + OA: Effect of differentiation of CPCs P4, followed by 1 week of OA 300 μM, on cell mitochondria (c), compared with CNTRL undifferentiated CPCs (a) and differentiated CPCs without OA supplementation (b); all stained with MitoTracker® Red CMXRos. (scale bars: 50 μm)

The TGF-β1 protocol increased gene expression of both PPARα and PGC-1α. Addition of OA for 1 week maintained the PPARα gene expression at the same level, but PGC-1α expression decreased (Fig. 7A). Glut1 gene expression was significantly downregulated after OA treatment, accompanied by a significant increase in Glut4 expression after TGF-β1 differentiation, which increased further following OA treatment. Pdk4 and Cs expression remained unchanged in both treatments (Fig. 7A). Assessment of the mitochondrial membrane potential, showed an increase in the fluorescence between the CNTRL undifferentiated CPCs, compared to the DIFF group and the DIFF+OA differentiated CPCs (Supplemental Fig. S5), (Fig. 7B).The glucose oxidation rates were increased after differentiation with TGF-β1, and afterwards further in differentiated cells treated with OA compared with rates in CNTRL undifferentiated cells (Fig. 7C). FA oxidation (Fig. 7E) increased in differentiated cells treated with OA, whereas rates of glycolytic flux which had been significantly increased by differentiation alone, were further elevated with OA treatment (Fig. 7D).

## 4 Discussion

To our knowledge, this is the first report that investigates the effect of fatty acid supplementation on adult cardiac progenitors, using metabolic flux analyses, focusing on optimising differentiation efficiency. Our results shed light on the metabolic profile of differentiating CPCs and highlight the importance of substrate availability, and the PPARα pathway, in this process.

Different groups have used the TGF-β1 family (Smits et al., 2009; Goumans et al., 2008; Ye et al., 2012) and 5-Aza (Oh et al., 2003; Qian et al., 2012; Goumans et al., 2008) on various cardiac cell types to induce cardiac differentiation *in vitro*. The methods of assessment of the level of CPC differentiation vary, but entail changes in cell morphology, as well as expression of markers such as Tnnt2, Myh7 and Cx43 (Goumans et al., 2008; Qian et al., 2012; Linke et al., 2005). Smits et al. (Smits et al., 2009) observed that cells formed multiple layers with different alignments and confluency, after TGF-β1 differentiation of selected Sca1^+^ CPCs, as well as upregulation in Tnnt2, Cx43 and Myh7, which agrees with our observation of cell culture morphology and marker expression (Fig. 1B and C). The pattern of MYH7 expression was concentrated within the tubular structures (Fig. 1D), suggesting that there was still a fraction of undifferentiated cells growing around the differentiating ones, which may indicate that cells need to be highly confluent for efficient differentiation. The main cell culture issue that we faced, had to do with the fact that the cells were difficult to dissociate as they formed clumps after the differentiation of month. Subsequently, the cells were trypsinised, before pelleting for RNA extraction, which did not work well with the cell clusters in the differentiated samples, which may have biased the analysis of gene expression to the easily-dissociated monolayer cells (Fig. 1C).

The upregulation of cardiac transcription factors, shown in studies of hESC differentiation (Kehat et al., 2004; Xu, 2002) and mESC (Sachinidis et al., 2003; Fijnvandraat et al., 2003) into CMs, as well as MHC and Troponin (Fijnvandraat et al., 2003; Van Laake et al., 2005), matched those seen in the differentiating mESCs at d4, d7 and d14. (Fig. 2A). The downregulation of Glut1 and upregulation of Glut4 observed has been reported as an indication of the metabolic maturation of cells (Wang and Hu, 1991; Studelska et al., 1992; Postic et al., 1994) (Fig. 2B). The early increase in PGC-1α could indicate upregulation of the mitochondrial metabolism early in the differentiating SCs (Chung et al., 2010; Porter et al., 2011; Lehman et al., 2000), followed by an increase in PPARα, as seen previously in murine cardiac differentiation *in vitro* (Ding et al., 2007b; Vega et al., 2000) (Fig. 2C). These observations suggested that the mESCs differentiating to beating CMs *in vitro* recapitulate the metabolic shift towards oxidative metabolism and express mature cardiac cell markers, rendering them a good control for this study.

After identifying the metabolic changes characterising successful differentiation of mESCs to beating CMs, we proceeded to study the metabolism of differentiating mouse adult CPCs. The upregulation of PPARα and PGC-1α after OA supplementation (Fig. 2C &3C) agrees with OA, and FAs in general, being activators of the PPARα pathway (Popeijus et al., 2014; Göttlicher et al., 1992). Pdk4, which has been shown to be upregulated by PGC-1α (Wende et al., 2005) was upregulated at 48 h, while CD36, which mediates the uptake of fatty acids in a variety of cell types (Pepino et al., 2014); Coburn et al., 2001), was upregulated at all time-points (Fig. 3B). The shift from Glut1 to Glut4 was similar to that seen with mESC differentiation, consistent with cell maturation (Wang and Hu, 1991; Studelska et al., 1992; Postic et al., 1994) (Figs. 3B & 2B). Also, OA treatment increased mitochondrial membrane potential (Figs. 4C & Supplementary Figs. S4 & S5) after 48 h and 1 week of treatment. The effect was on mitochondrial activity, rather than the mitochondrial numbers, since CS was found unchanged – both of which are necessary for cell maturation (Porter et al., 2011).

Glucose oxidation was upregulated significantly, while glycolytic flux decreased, after 1 week of OA treatment, suggesting a shift towards a more mature metabolic phenotype, but FA oxidation rates were unchanged (Fig. 5A, B and C). This is the first study assessing the metabolism of differentiating adult CPCs *in vitro,* so there is no prior evidence of the expected metabolic profile. It may be that the OA-treated cells required a longer supplementation period in order to significantly upregulate FA oxidation, or that the undifferentiated cells obtained sufficient energy from glucose oxidation.

To investigate whether the metabolic effect that followed the OA treatment could assist the differentiation of adult CPCs, the cell culture medium was supplemented for 1 week following the TGF-β1 differentiation protocol. The gene expression changes of the OA-treated differentiated CPCs revealed a more structurally mature phenotype, with both Myh7 and Cx43 being significantly upregulated (Fig. 6B). In addition, TGF-β1 differentiation alone was sufficient to stimulate the PPARα – PGC-1α pathway, as well as a shift from Glut1 to Glut4 gene expression (Fig. 7A), as seen during the mESC differentiation previously. PPARα and PGC-1α are upregulated during differentiation (Ding et al., 2007b), and the expression of GLUT4 is higher in adult tissues (Wang and Hu, 1991; Studelska et al., 1992; Postic et al., 1994), suggesting that TGF-β1 differentiation induces a degree of metabolic maturation.

Addition of OA after differentiation retained the levels of PPARα, Glut1 and Glut4, but led to a reduction in PGC-1α (Fig. 7A), a decrease that was also seen at d14 of mESCs progressing to terminal differentiation (Fig. 2C), and after long-term exposure of CNTRL cells with OA (Fig. 4B). This could suggest that PGC-1α does not need to be constitutively upregulated after changes have been induced, although Birket et al. reported consistent activation of PGC-1α in ESCs during cardiomyocyte differentiation (Birket et al., 2013).

Glucose and FA oxidation, as well as glycolytic flux, were significantly upregulated in the TGF-β1 differentiated CPCs treated with OA (Fig. 7C, D and E), indicating a holistic metabolic maturation of the differentiated cells after OA supplementation. The TGF-β1 differentiated CPCs seem to acquire a more mature metabolic phenotype (because of the pharmacological agent), and respond differently to undifferentiated CPCs when OA is added, by upregulating FA oxidation. The fact that OA supplementation had a positive effect in the maturation of the differentiated CPCs came as no surprise, given the recent study from Correia et al. (Correia et al., 2017) that demonstrated the importance of the metabolic shift in the maturation of hPSC-CMs. The hPSCs that were supplemented with FAs had enhanced differentiated profile with increased oxidative metabolism and functional maturation, compared to those grown in the standard glucose media, a finding that agrees with our observations. In this study the FA treatment entailed combination of both oleic and palmitic acids, as well as galactose, whereas we only used oleic acid, but it would be interesting to explore a better combination of substrates that can enhance cardiac differentiation to mature CMs from adult cardiac progenitors.

## 5 Conclusions

This is the first study that uses radiolabelled substrate consumption for metabolic flux analyses of adult cardiac progenitors, after FA supplementation, with the aim to enhance their cardiac differentiation. In addition, we explore the role of the PPARα/PGC-1α axis during metabolic manipulation.

We have shown that OA supplementation matured both control and differentiated cells metabolically, while also increased the expression of cardiac markers in the latter. In addition we highlight that the PPARα pathway was stimulated by cardiac differentiation in both CPCs and mESCs. Our results allow for a better understanding of the effect of fatty acids, on cardiac differentiation of adult cardiac progenitors, complementing the metabolic manipulation studies of hPSC-CMs, and development of a protocol that would allow for more efficient differentiation to mature CMs.

The following are the supplementary data related to this article.

**Fig. S6:**
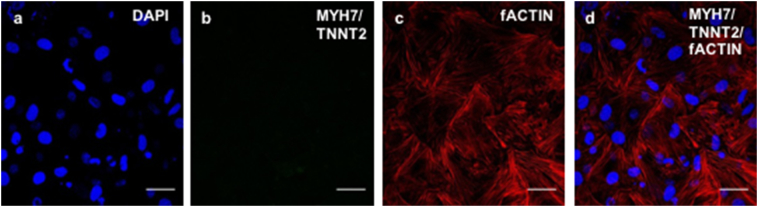
Negative control staining for differentiation imaging: Negative control staining with secondary antibodies for immunocytochemistry of MHC and TnnT2 on differentiated CPCs with the TGF-β1 protocol (see Fig. 6C), co-stained with DAPI in blue (scale bars: 50 μm)

Supplementary material Image 1
