# Supplementary material for: Metabolic flux analyses to assess the differentiation of adult cardiac progenitors after fatty acid supplementation
Source: Stem Cell Res. 2019 Jul;38:101458. doi: 10.1016/j.scr.2019.101458 (PMC6618003; doi:10.1016/j.scr.2019.101458)
Supplement: Supplementary file 1 — Supplementary material [file mmc1.docx]

**SUPPLEMENTARY MATERIAL**

**9.1 SUPPLEMENTARY METHODS**

9.1.1 CHARACTERISATION OF CARDIAC PROGENITOR CELLS

CPCs were isolated from mouse atria by a collagenase/trypsin digestion, followed by a slow adhesion period of 48 hours, subsequently were passaged and used at P4 for all experiments. mRNA expression in CPCs was compared to mESCs differentiating to CMs, through embryoid body formation (EBs) at both d4 and d7 of differentiation. CPCs had similar expression of MEF2C and GATA4 but lower expression of the stemness markersOCT3/4, CKIT and TERT, with EBs at day 7 of differentiation (Supplemental figure S2 B). Immunocytochemistry results indicated the expression of Sca1 and CD90 markers (Supplemental figure S2 C)

**9.2 SUPPLEMENTARY MATERIAL**

## 9.2.1 RT-PCR PRIMERS

A custom-made primer-array for cardiac progenitor markers (Gata4, Nkx2.5, Ckit, Isl1, Mef2c, Flk1) was designed and validated by PrimerDesign (Southampton, UK). All the other primers used in this study were designed using the NCBI Primer-Blast online tool ([www.ncbi.nlm.nih.gov/tools/primer-blast/](http://www.ncbi.nlm.nih.gov/tools/primer-blast/)).

| **Target gene** | **Sequence 5’-3’** | **Supplier** |
| --- | --- | --- |
| Glut1 Forward | CTGGCGGGAGACGCATAGTT | Sigma Aldrich |
| Glut 1 Reverse | GAACTCCTCAATAACCTTCTGGGG | Sigma Aldrich |
| Glut 4 Forward | GGCTCTGACGATGGGGAACC | Sigma Aldrich |
| Glut 4 Reverse | AAACTGAAGGGAGCCAAGCA | Sigma Aldrich |
| Pgc1α Forward | TCTCAGAAGGGGCTGGTTG | Sigma Aldrich |
| Pgc1α Reverse | AGCAGCACACTCTATGTCACT | Sigma Aldrich |
| Pparα Forward | ACTACGGAGTTCACGCATGTG | Sigma Aldrich |
| Pparα Reverse | TTGTCGTACACCAGCTTCAGC | Sigma Aldrich |
| Tnnt2 Forward | CTGAGACAGAGGAGGCCAAC | Sigma Aldrich |
| Tnnt2 Reverse | ACCAAGTTGGGCATGAAGAG | Sigma Aldrich |
| Cs Forward | TCCATCACAGCGGCGAC | Sigma Aldrich |
| Cs Reverse | AGGCAGGATGAGTTCTTGGC | Sigma Aldrich |
| Hprt Forward | TCAGTCAACGGGGGACATAA | Sigma Aldrich |
| Hprt Reverse | GGGGCTGTACTGCTTAACCAG | Sigma Aldrich |
| Sdha Forward | AACTACAAGGGACAGGTGCTG | Sigma Aldrich |
| Sdha Reverse | CTCCCACAGGCATACAGAC | Sigma Aldrich |
| Pdk4 Forward | CAAAGACGGGAAACCCAAGC | Sigma Aldrich |
| Pdk4 Reverse | CACACTCAAAGGCATCTTGGAC | Sigma Aldrich |
| Oct3/4 Forward | GAGAACCGTGTGAGGTGGAG | Sigma Aldrich |
| Oct3/4 3 Reverse | TCGAACCACATCCTTCTCTAGC | Sigma Aldrich |
| Wt1 Forward | TTCAAGGACTGCGAGAGAAG | Sigma Aldrich |
| Wt1 Reverse | GGGAAAACTTTCGCTGACAA | Sigma Aldrich |
| Myh7 2 Forward | CTACCAGACAGAGGAAGACAGGA | Sigma Aldrich |
| Myh7 2 Reverse | TTGGAGCTGGGTAGCACAAGA | Sigma Aldrich |
| Cd36 Forward | TTAATGGCACAGACGCAGCC | Sigma Aldrich |
| Cd36 Reverse | GGATTCTGGAGGGGTGATGC | Sigma Aldrich |
| Vimentin Forward | CAGCAGTATGAAAGCGTGGC | Sigma Aldrich |
| Vimentin Reverse | CAGAGAGGTCAGCAAACTTGG | Sigma Aldrich |
| Cx43 Forward | GAAGTACCCAACAGCAGCAG | Sigma Aldrich |
| Cx43 Reverse | TGGGCACCTCTCTTTCACTTAAT | Sigma Aldrich |
| CD105 Forward | GGTACAGTGCATCGACATGG | Sigma Aldrich |
| CD105 Reverse | CAGAATCCCACAAGCTCCAA | Sigma Aldrich |
| Ddr2 Forward | ACTACAGTCGGGATGGCAAC | Sigma Aldrich |
| Ddr2 Reverse | ACACGTTCATGGAGTGGTCA | Sigma Aldrich |
| CD90 Forward | CAGAATCCCACAAGCTCCAA | Sigma Aldrich |
| CD90 Reverse | GCCAGGAAGTGTTTTGAACC | Sigma Aldrich |
